# Pulmonary Rehabilitation Exercise Package for Enhancing Health in Elderly COPD Patients With Frailty: An Experimental Study

**DOI:** 10.1097/jnr.0000000000000698

**Published:** 2025-09-04

**Authors:** Lin-Yu Liao, Huan-Hwa Chen, Fenju Chen, Shunt-Chen Yang

**Affiliations:** 1 Department of Nursing, Chest Hospital, Ministry of Health and Welfare, Tainan, Taiwan; 2 Department of Nursing, Chung Hwa University of Medical Technology, Tainan, Taiwan; 3 Department of Health Administration, I-Shou University, Kaohsiung, Taiwan; 4 Department of New Star Yu Home Nursing Center, Kaohsiung, Taiwan

**Keywords:** COPD, frail, pulmonary rehabilitation exercise

## Abstract

**Background:**

Frailty may result in decreased physical functioning and worsen the prognosis of chronic diseases. Chronic obstructive pulmonary disease (COPD) is associated with an increased risk of concurrent frailty. Although pulmonary rehabilitation has demonstrated improvements in COPD outcomes, its impact on patients with frailty and COPD remains unclear.

**Purpose:**

This study was designed to examine the effects of the pulmonary rehabilitation exercise package (PREP) on frailty, dyspnea, lower extremity muscular endurance (LEME), and walking ability (WA) in older adult COPD patients with frailty.

**Methods:**

A single-blind experimental design was used to study 100 elderly COPD patients with frailty, randomly assigned to either the experimental or control group. The experimental group (EG) received the PREP intervention, while the control group (CG) received routine care. The Clinical Frail Scale (CFS) was used to measure frailty, the Modified Medical Research Council scale was used to measure dyspnea, LEME was measured using the 30-second chair stand test, and functional exercise capacity (i.e., walking ability or WA) was measured using the 6-minute walk distance. All measurements were taken at three time points: baseline (preintervention), 1 week postintervention, and 1 month postintervention. Between-group and within-group differences and variations in repeated measurements over time were compared using independent *t* tests, paired *t* tests, and generalized estimating equations (GEE).

**Results:**

A total of 91 participants completed the study, with 9 participants lost to follow-up. No significant between-group differences were found at baseline in terms of characteristics, frailty, dyspnea, LEME, and WA. Applying difference-in-differences, the EG outperformed the CG in terms of dyspnea and WA at both 1-week and 1-month follow-ups, while the EG significantly outperformed the CG on all measures at the 1-month follow-up. Within-group comparisons also revealed significant improvements in the EG compared with the CG. Using GEE to examine the interaction, the EG demonstrated significantly better improvements in dyspnea, LEME, and WA than the CG at the 1-month mark.

**Conclusions/Implications for Future Practice:**

The results show that PREP has the potential to significantly improve health in older adults with frailty and COPD by addressing frailty, dyspnea, LEME, and WA. PREP may be implemented as a subacute health care model to manage COPD-related debilitation in hospital settings.

## Introduction

Frailty and chronic obstructive pulmonary disease (COPD) are crucial health concerns that significantly impact the well-being of sufferers. Frailty exacerbates COPD symptoms through several mechanisms, including decreased muscle strength and reduced physical activity, which in turn worsen dyspnea and fatigue. In addition, frailty may increase inflammatory responses and decrease immune function, further exacerbating COPD progression (Kennedy et al., 2019). Patients with both frailty and COPD face higher risks of disability and mortality (Prince et al., 2015; Proietti & Cesari, 2020). Older adults, particularly those over 65, face a substantial risk of frailty (Alqahtani et al., 2021; Filho et al., 2020). Also, inpatients with pulmonary diseases, including COPD, may be at risk of frailty across all ages, though older adults are more likely to experience adverse outcomes (Singer et al., 2016). In Taiwan’s rapidly aging population, frailty has become a pressing public health concern. Recent studies report that 16.9% of community-dwelling older adults are frail, while over 54% of hospitalized seniors are at moderate-to-high frailty risk (Chen et al., 2024; Chen et al., 2025). This has prompted the country’s National Health Service to prioritize the provision of frail care and rehabilitation programs in hospitals to prevent disability.

Frailty, often concomitant with chronic diseases, is notably higher in older individuals with COPD than their peers without COPD (10.2% vs. 3.4%; Lahousse et al., 2016). Remarkably, 85% of COPD patients are susceptible to frailty (Beek et al., 2020). Detecting frailty in older COPD individuals is challenging due to shared symptoms such as weakness, fatigue, unintentional weight loss, slow walking speed, reduced grip strength, and increased susceptibility to illness and injury (Proietti & Cesari, 2020). COPD involves chronic respiratory tract inflammation, leading to symptoms such as shortness of breath, chronic cough, wheezing, fatigue, and reduced exercise tolerance (Rahi et al., 2023). In the literature, COPD is identified as a respiratory disease that affects the lungs, while frailty is a syndrome associated with aging. Although two different medical concepts, these two conditions share certain similarities, especially in older adult patients, in terms of symptoms, including dyspnea, fatigue, reduced physical capacity, decreased mobility, poor appetite, and weight loss; psychological issues such as anxiety, depression, and social isolation; multiple chronic conditions, and a decreased quality of life (Kennedy et al., 2019). Thus, based on prior reporting, a bidirectional relationship may exist between COPD symptoms and frailty. While the symptoms of COPD may negatively impact physical fitness, leading to or exacerbating frailty, frailty increases the risk of developing and worsening COPD symptoms. The relationship between these two conditions is mutually influential and aggravating.

Frailty increases the risk of developing COPD and reduces muscle strength, weakening the respiratory muscles, making breathing more difficult, and exacerbating dyspnea (shortness of breath). The weakening of other muscles (e.g., those in the limbs) restricts daily activities, making it harder for patients to perform basic tasks such as walking and climbing stairs. Reduced physical activity further impacts cardiopulmonary function, worsening dyspnea, and fatigue (Kennedy et al., 2019). Moreover, frailty may increase inflammatory responses and decrease immune function. Often accompanied by chronic low-grade inflammation, frailty may thus lead to elevated levels of inflammatory markers such as C-reactive protein and interleukin-6. COPD itself is an inflammatory disease, and frailty-associated inflammation may further exacerbate airway and lung tissue inflammation, worsening the condition (Kennedy et al., 2019; Lahousse et al., 2016). Furthermore, frailty weakens the immune system, reducing the body’s ability to resist infection, making patients with COPD more susceptible to viral or bacterial infections, worsening COPD symptoms, and leading to acute exacerbations. Declines in immune function also affect the body’s ability to repair and recover, making it more difficult for these patients to recover from acute exacerbations, worsening the condition even more (Yan et al., 2023).

Exercise is recommended for older individuals with frailty to counteract the associated decline in physical fitness (Navarrete-Villanueva et al., 2021). It has been proven to reduce age-related oxidative damage, chronic inflammation, and enhance various physiological factors such as mitochondrial function, actin profile, insulin-like growth factor-1 signaling, and insulin sensitivity (Angulo et al., 2020). Specifically, engaging in pulmonary rehabilitation exercises has been reported to have positive effects on symptom management and quality of life in patients with chronic respiratory diseases (Vitacca et al., 2017). Also, prior findings indicate pulmonary rehabilitation exercises have a significant enhancement effect on frailty, primarily by enhancing lung function, increasing muscle strength, and improving ventilation and gas exchange, leading to increased overall oxygen uptake capacity (Finamore et al., 2021). Respiratory rehabilitation exercises include pursed-lip breathing, which can strengthen respiratory muscles, enhance oxygen supply, and improve carbon dioxide elimination. Postural drainage combined with chest percussion accelerates sputum expulsion and alleviates dyspnea. Bed exercises such as leg lifts, toe raises, and foot flexion can strengthen the quadriceps and calf muscles, improving walking ability and daily activity performance (Attwell & Vassallo, 2017; Beaumont et al., 2018; Liao et al., 2020).

Current research explores the application of pulmonary rehabilitation exercises in patients with frailty and stable COPD, emphasizing community settings to reduce frailty and enhance muscle endurance and exercise tolerance (Attwell & Vassallo, 2017; Maddocks et al., 2016). Frailty prevalence among COPD patients has been previously investigated (e.g., Beek et al., 2020; Lahousse et al., 2016). Respiratory rehabilitation in elderly patients with frailty and stable COPD has been shown to enhance physical activity levels and alleviate dyspnea (Maddocks et al., 2016), reduce frailty severity (Mittal et al., 2015), increase overall oxygen uptake, and alleviate fatigue (Finamore et al., 2021). However, prior research into this issue has focused primarily on the community-dwelling population with COPD and frailty, which tends to have more stable disease characteristics. The exercise programs in these studies emphasize physical activity rather than respiratory training. However, the interaction between frailty and interdisciplinary pulmonary rehabilitation exercises in older adult patients with unstable conditions, such as frailty and COPD, has not been explored. Specifically, the impact of rehabilitation interventions on patients undergoing pulmonary rehabilitation during hospitalization remains undiscussed. The primary goal of the pulmonary rehabilitation exercise package (PREP) is to address frailty directly. By focusing on improving dyspnea, lower extremity muscular endurance (LEME), and walking ability (WA), PREP may be applied to reduce the severity of frailty in older individuals with COPD. Therefore, in this study, engaging in PREP is hypothesized to yield significant improvements in frailty, dyspnea, LEME, and WA in older individuals with COPD.

## Methods

### Study Design

A single-blind experimental design with random assignments for the experimental and control groups was employed in this study. Participants with even-numbered medical record numbers were assigned to the experimental group (EG), and those with odd-numbered medical record numbers were assigned to the control group (CG), with EG and CG participants, respectively, assigned to rooms in the east and west wings of the hospital to ensure the transparency and reliability of the research methodology and results. Both groups received routine treatment, with the EG receiving an additional comprehensive pulmonary rehabilitation exercise package (PREP) and the CG participating in regular activities. A single-blind design was used to minimize learning effects by collecting data first from the EG and afterward from the CG. Data on frailty, LEME, and WA were assessed at baseline, 1 week, and 1 month after the intervention and collected from January 1 to October 30, 2020, at a thoracic specialist hospital in southern Taiwan.

### Participants

Frailty in the participants was assessed using the Clinical Frailty Scale (CFS), which is scored on a 9-degree scale ranging from “very fit” to “terminally ill,” with scores between 4 and 6 indicating moderate to high frailty. The CFS has proven effective in evaluating the overall functional and health status of older adults in many prior studies. Participants were eligible for inclusion if they had a physician-confirmed diagnosis of COPD, demonstrated mental clarity, were able to communicate in either Mandarin or Taiwanese, and provided written informed consent after receiving a comprehensive explanation of the study’s objectives and procedures. Exclusion criteria included having unstable vital signs, having a blood oxygen concentration below 90%, and being unable to be mobile or walk.

The minimum sample size for this study was 84, as determined using G*Power 3.1.9.2 software with an effect size of 0.80, α error probability of .05, and statistical power of 0.95. Considering a 20% potential loss to follow-up, 100 participants were recruited.

### Ethical Considerations

The study was approved by the Jianan Psychiatric Center, Ministry of Health and Welfare, Taiwan (IRB No: 19-041). Prospective participants were given a detailed informed consent document before enrollment. The researcher explained the study’s purpose, process, acceptance conditions, exclusion criteria, and intervention procedures. The participants were also informed of their right to withdraw at any time and were enrolled upon signing informed consent.

### Pulmonary Rehabilitation Exercise Package

The PREP intervention used in this study is based on a protocol described by Liao et al. (2015) and includes the following components:

1.
***Disease Education:*** The participants were educated about lung disease signs using detailed anatomy diagrams that were reviewed twice daily in 30-minute educational sessions.
2.
***Postural Drainage and Lung Percussion:*** Family members received a card illustrating related techniques. The participants practiced these techniques twice daily in the 30-minute education sessions.
3.
***Pursed-Lip Breathing:*** A trainer led the participants in practicing this exercise at least twice daily for 2 minutes each session.
4.
***Leg-Raising Exercise on the Bed:*** Twice daily for 5 minutes per session, the participants lifted each foot 5 cm from the bed and repeated the exercise at least five times for each foot.
5.
***Calf Muscle Strengthening:*** The participants performed this exercise at least twice daily for 5 minutes per session, repeating each recommended move 10 times.
6.
***Additional Care Services:*** The participants received discharge preparations and long-term care services. A comprehensive care needs assessment was performed at discharge. The participants returned home within 3 days of hospitalization, and the implementation of the care plan was evaluated daily by a manager during hospitalization.

### General Care

The routine care provided in this study was supervised by the primary nurse and involved various components to support patient well-being. These components included disease education, upper limb exercise rehabilitation, guidance on using hand-held sprayers, nutrition health education, smoking cessation advice, and sputum cleaning skills.

### Measurements

#### Demographics

The demographic data collected in this research include age, gender, height, weight, body mass index (BMI), educational level, marital status, smoking status, and drinking status. In addition, pulmonary function was measured using the forced expiratory volume in 1 second/forced vital capacity (FEV1/FVC) ratio. A normal FEV1/FVC ratio is defined as >0.7, with lower values indicating poorer lung function. The Charlson Comorbidity Index (CCI) was also assessed to evaluate the presence and severity of comorbidities, assigning higher scores to individuals with a greater number and more severe comorbid conditions.

#### Frailty Measurement

The frailty measurement used in this study was the Chinese version of the Clinical Frail Scale (CFS), which has shown high reliability and validity in diverse clinical settings both before and uring hospitalization (Moloney et al., 2023). The CFS is a commonly used tool for assessing frailty, measuring the overall functional status and health of older adults (Chan & Pin, 2019). The total CFS score range is 1–9, with 1 indicating very fit (robust, energetic, no chronic illnesses, capable of high-intensity physical activity), 2 indicating well (healthy, no significant health issues, but not as vigorous as 1), 3 indicating managing well (has chronic diseases, well-controlled, no apparent functional limitations), 4 indicating vulnerable (mildly frail, some symptoms or chronic diseases, but can independently perform daily activities), 5 indicating mildly frail (more noticeable frailty, needs help with some daily activities), 6 indicating moderately frail (obvious frailty, needs help with most daily activities), 7 indicating severely frail (severe frailty, needs almost complete care), 8 indicating very severely frail (nearly completely dependent on others, critically ill), and 9 indicating terminally ill (bedbound, limited life expectancy). A CFS score >6 generally indicates moderate to severe frailty (Wharton et al., 2019). Hence, in this study, a CFS score between 4 and 6 was included as an inclusion criterion to ensure that, based on feasibility and ethical considerations, the participants were able to participate in the study without being too frail to engage or bearing additional risks. The accuracy of the Clinical Frailty Scale (CFS), as reported in a meta-analysis of 13 studies by Moloney et al. (2023), demonstrated high diagnostic performance in acute care settings. The overall accuracy was 0.89 (95% CI [0.86, 0.90]), indicating that the CFS is a reliable and valid tool for identifying frailty in unstable or hospitalized older adults.

#### Dyspnea

The modified Medical Research Council (mMRC) was used in this study to assess dyspnea status (Beaumont et al., 2018; Munari et al., 2018). The 5-item mMRC scale is scored from 0 to 5, with higher scores indicating greater severity of dyspnea impact. A score of 0 indicates dyspnea during intense exercise only, 1 indicates shortness of breath during brisk walking or ascending a slight slope, 2 indicates slower walking pace or pausing to catch one’s breath on flat surfaces, 3 indicates the need to stop and catch one’s breath every few minutes after walking ∼100 m on flat surfaces, and 4 indicates the inability to go outdoors or feeling breathless while dressing and undressing (Richards, 2017). The mMRC in this study demonstrated good validity, supported by a correlation coefficient of .76 (Yamaguchi et al., 2021). The Cronbach’s α in this study was .67.

#### Lower Extremity Muscular Endurance

The 30-second Chair Stand Test (30s-CST) was employed to measure the number of standing up and sitting down movements the participants could perform within a 30-second timeframe, with the result serving as an indicator of lower limb muscle strength and more repetitions indicating greater strength. The 30s-CST is widely used in assessing lower limb muscle strength in hospitalized older individuals during the acute phase (Liao et al., 2020). The participants were instructed to sit in the center of the chair, cross their hands in front of their chest, and, upon hearing the command “Start,” stand up using both legs with fully extended knees before returning to a seated position. This measure achieved an intraclass correlation coefficient of .99 in a prior evaluation of lower extremity muscle strength of older individuals with COPD (Liao et al., 2020) and in this study earned a Cronbach’s α for internal consistency of .95.

#### Walking Ability (WA)

In this study, walking ability was assessed using the 6-minute walking distance (6MWD) test conducted in a 30-m-long level corridor (Agarwala & Salzman, 2020). The 6MWD measures the distance an individual can walk in 6 minutes, with longer distances indicating better walking ability and exercise tolerance (Bohannon & Crouch, 2017; Kerti et al., 2018). In prior reliability testing, the inter-rater reliability for assessing cardiorespiratory endurance and walking ability in frail older individuals has ranged from .86 to .96 (Bohannon & Crouch, 2017; Kerti et al., 2018). Also, this test earned an intraclass correlation coefficient of .99 in an assessment of older individuals with COPD (Liao et al., 2020) and a Cronbach’s α of .97 in this study.

### Data Collection

Data for pretest, 1-week posttest, and 1-month posttest were collected by the researcher. The participants in both groups were identified using the hospital’s inpatient and outpatient management systems. On the day of admission, the researcher reviewed the medical records of each participant to gather the targeted demographic and disease data. Pretests for dyspnea, frailty, and walking distance were conducted in the ward for both groups and the participants and their families were reminded about the follow-up outpatient visits at 1 week and 1 month postdischarge, when posttest data would be collected. The CFS was used in this study to ensure the accuracy of frailty assessments (Moloney et al., 2023), and the assessors were required to have at least N3 level qualifications and to have attended the 2-hour annual training class on frailty-related knowledge and assessment techniques. To minimize variability in the assessment process and ensure consistency, the principal investigator reviewed the assessment results after each evaluation. If any issues were identified, further communication and adjustments were made.

To monitor adherence to the intervention, discharge preparation service case managers provided routine care to the control group and standardized PREP guidance leaflets to the experimental group. This continued until the participants were discharged, with ongoing follow-up and guidance conducted via phone calls and video consultations. The researcher did not participate in the PREP guidance but regularly discussed the implementation of intervention measures with the case managers to ensure they were carried out properly.

To enhance methodological transparency, the participants were assigned to experimental and control groups randomly based on their respective medical record numbers, with those with even numbers assigned to the experimental group and those with odd numbers assigned to the control group. To prevent cross-contamination and ensure blinding, the experimental group was placed in the east wing of the hospital, and the control group was placed in the west wing. One hundred patients meeting the inclusion criteria (*N*=100) were enrolled as participants, and vital signs, finger blood oxygenation, and medical history were assessed for all. Basic participant information, physiological indices, and medical conditions were collected. Four were excluded due to unstable vital signs, low blood oxygen levels (<90%), or inability to mobilize. Thus, 96 participants were randomly assigned to the experimental or control group. Initial assessments of dyspnea, LEME, and WA were conducted.

**Figure 1:**
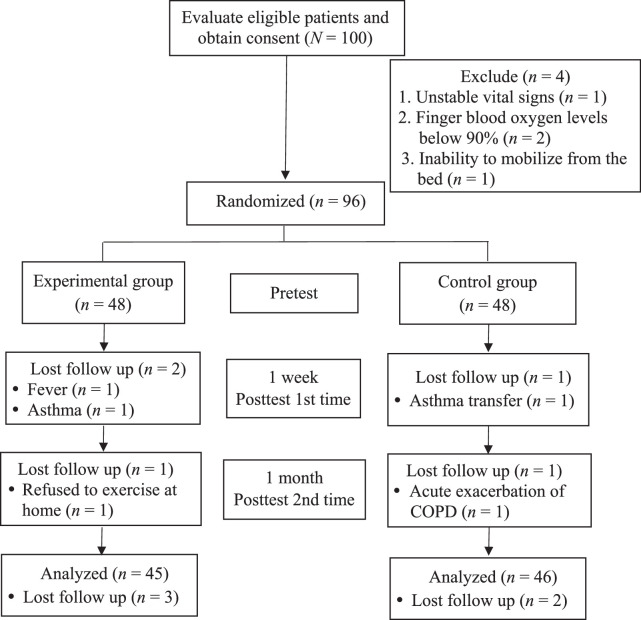
Flowchart of the Participant Selection Process

In terms of the outcome assessment, the evaluators were blinded to group assignments to avoid assessment bias. The experimental group underwent a 1-month PREP program and the control group received routine care. Postdischarge, telephone care was used in monitoring exercise adherence. After 1-week, medical records were checked, and a second (first posttest) assessment was conducted, with three participants lost to follow-up. At the second posttest assessment conducted during the 1-month clinical visit, a further two patients were lost to follow-up because one refused to do the home exercise and the other had experienced COPD exacerbation requiring readmission (Figure 1).

### Statistical Analysis

After retrieving, encoding, and inputting data into the computer, thorough verification was performed. Data analysis was conducted using IBM SPSS Statistics 22.0 (IBM Corp., Armonk, NY, USA), a statistical software for Windows in the Chinese version. Descriptive statistics, including frequency distribution, percentages, means, and SDs, presented the basic characteristics of the research subjects. The χ^2^ test and independent samples *t* test assessed demographic homogeneity between the groups. The generalized estimating equation (GEE) examined the interaction between groups and time for dyspnea, LEME, and WA. A significance level of *p*<.05 was considered statistically significant, indicating a meaningful finding.

## Results

### Characteristics of Participants

Ninety-one participants successfully completed the study, with 45 in the experimental group (*n*=45) and 46 in the control group (*n*=46). In terms of the sample, the average age was 70.04 (*SD*=11.29) years, the average BMI was 23.16 (*SD*=4.85) kg/m^2^, the average CCI score was 4.34 (*SD*=1.59), the average FEV1/FVC ratio was 66.53% (*SD*=14.45), and the average frailty score was 4.65 (*SD*=0.96). In terms of gender distribution, most of the participants were male (81.22%, *n*=74) and married (71.43%, *n*=65). Regarding education, 48.35% (*n*=44) had an elementary school education. The majority reported no religious beliefs (58.24%, *n*=53), were nonsmokers (71.43%, *n*=65), and nondrinkers (90.11%). Self-assessed health status resulted in half (49.45%; *n*=45) rating their health as poor, followed by 43.96% (*n*=40) reporting their health as neither good nor bad. The results of the independent sample *t* tests and χ^2^ tests showed no significant differences between the groups (*p*>.05; Table 1).

**Table 1:** Participant Demographics (*N*=91)

Variable	*M* (*SD*)			*t/*χ^2^	*p*
	*N*=91	EG (*n*=45)	CG (*n*=46)		
Age (year)	70.04 (11.29)	70.80 (10.84)	69.30 (11.79)	−0.63	.531
BMI (kg/m^2^)	23.16 (4.85)	22.36 (4.03)	23.94 (5.47)	1.57	.120
Comorbidities ^a^	4.34 (1.59)	4.64 (1.71)	4.04 (1.41)	-1.83	.071
FEV1/FVC (%)	66.53 (14.45)	64.43 (12.81)	68.59 (15.76)	1.38	.171
	*n* (*%*)	*n* (*%*)	*n* (*%*)		
Gender				3.83	.064
Male	74 (81.22)	33 (73.33)	41 (89.13)		
Female	17 (18.78)	12 (26.67)	5 (10.87)		
Marital status				2.16	.539
Unmarried	6 (6.59)	2 (4.44)	4 (8.70)		
Married	65 (71.43)	35 (77.78)	30 (65.22)		
Divorce	9 (9.89)	3 (6.67)	6 (13.04)		
Widowed	11 (12.09)	5 (11.11)	6 (13.04)		
Education				0.21	.976
Illiterate	9 (9.89)	4 (8.89)	5 (10.87)		
Elementary school	44 (48.35)	22 (48.89)	22 (47.83)		
Junior high school	15 (16.48)	8 (17.78)	7 (15.21)		
≥ Senior high school	23 (25.27)	11 (24.44)	12 (26.09)		
Religion				2.61	.138
Yes	38 (41.76)	15 (33.33)	23 (50)		
No	53 (58.24)	30 (66.67)	23 (50)		
Smoking				0.16	.817
Yes	26 (28.57)	12 (26.67)	14 (30.43)		
No	65 (71.43)	33 (73.33)	32 (69.57)		
Drinking				3.21	.073
Yes	9 (9.89)	7 (15.56)	2 (4.35)		
No	82 (90.11)	38 (84.44)	44 (95.65)		
Self-rated health status				4.95	.176
Very poor	5 (5.49)	4 (8.89)	1 (2.17)		
Poor	45 (49.45)	25 (55.55)	20 (43.48)		
Not too bad	40 (43.96)	16 (35.56)	24 (52.17)		
Well	1 (1.10)	0 (0.00)	1 (2.17)		
Very well	0 (0.00)	0 (0.00)	0 (0.00)		

*Note*. EG=experimental group; CG=control group; BMI=body mass index; FEV1=forced expiratory volume in 1 second; FVC=forced vital capacity.

^a^ Comorbidity was measured using the Charlson comorbidity index.

### Between-Group and Within-Group Differences in Frailty, Dyspnea, Lower Extremity Muscular Endurance, and Walking Ability

As shown in Table 2, the between-group differences in terms of frailty, dyspnea, LEME, and WA at pretest were all insignificant (*p*>.05). At the first posttest (1 wk postintervention), the experimental group had significantly lower mean dyspnea than the control group (2.51 ± 0.73 vs. 2.83 ± 0.57; *p*=.024), and at the second posttest (1 month postintervention), this difference had increased further (i.e., 2.20 ± 0.63 vs. 2.52 ± 0.69; *p*=.022). However, at both posttest time points, none of the other parameters showed significant between-group differences (*p*>.05).

**Table 2:** Between-Group Comparisons of Frailty, Dyspnea, Lower Extremity Muscular Endurance, and Walking Ability at Pretest, 1-Week, and 1-Month (*N*=91)

Variable	*M* (*SD*)		*t*	*p*
	Experimental Group (*n*=45)	Control Group (*n*=46)		
Frailty ^a^				
Pretest	4.71 (0.89)	4.59 (1.02)	−0.62	.540
Posttest (1-week)	4.53 (0.89)	4.30 (0.87)	−1.24	.218
Posttest (1-month)	4.04 (1.13)	4.28 (0.91)	1.11	.270
Dyspnea ^b^				
Pretest	3.16 (0.64)	2.98 (0.58)	−1.39	.168
Posttest (1-week)	2.51 (0.73)	2.83 (0.57)	2.30	.024
Posttest (1-month)	2.20 (0.63)	2.52 (0.69)	2.33	.022
LEME ^c^				
Pretest	6.04 (4.22)	6.20 (3.68)	0.18	.856
Posttest (1-week)	6.60 (4.10)	6.98 (4.13)	0.44	.662
Posttest (1-month)	8.33 (4.05	7.24 (4.03)	−1.29	.200
Walking ability ^d^				
Pretest	126.98 (89.44)	135.87 (92.80)	0.47	.643
Posttest (1-week)	161.20 (84.73)	138.85 (88.47)	−1.23	.222
Posttest (1-month)	178.40 (94.04)	154.70 (93.96)	−1.20	.232

^a^ Frail was measured by the Clinical Frail Scale; ^b^ Dyspnea was measured by the Modified Medical Research Council; ^c^ LEME was measured by the 30-second chair stand test; ^d^ Walking ability was measured by the 6-minute walking distance.

As shown in Table 3, dyspnea, LEME, and WA improved significantly more in the experimental group compared with the control group between baseline and 1-week posttest (*p*<.05). Similarly, frailty, dyspnea, LEME, and WA improved significantly more in the experimental group compared with the control group between baseline and 1-month posttest (*p*<.05). In terms of within-group comparisons at both 1-week and 1-month intervals, the experimental group showed significant progress in frailty, dyspnea, LEME, and WA (*p*<.05), while the control group achieved significant improvements in dyspnea and WA only (*p*<.05). Notably, improvements in frailty, although present in both groups, did not reach statistical significance (*p*>.05).

**Table 3:** Between-Group and Within-Group Comparisons of Frailty, Dyspnea, Lower Extremity Muscular Endurance, and Walking Ability at Pretest, 1-Week, and 1-Month (*N*=91)

Variable	*M* (*SD*)		*t*	*p*
	Experimental Group (*n*=45)	Control Group (*n*=46)		
Frailty ^a^				
Pretest vs. posttest (1-week)	0.18 (0.49)	0.28 (0.66)	0.86	.391
Pretest vs. posttest (1-month)	0.67 (0.90)	0.30 (0.55)	−2.31	.023
Pair *t* test	−1.66 (*p*=.103)	0.22 (*p*=.830)		
Dyspnea ^b^				
Pretest vs. posttest (1-week)	0.64 (0.86)	0.15 (0.51)	−3.33	.001
Pretest vs. posttest (1-month)	0.96 (0.74)	0.46 (0.62)	−3.49	.001
Pair *t* test	-4.96 (*p*<.001)	−3.50 (*p*=.001)		
LEME ^c^				
Pretest vs. posttest (1-week)	−0.56 (1.41)	−0.78 (1.80)	−0.67	.505
Pretest vs. posttest (1-month)	−2.29 (2.37)	−1.04 (2.54)	2.42	.018
Pair *t* test	5.67 (*p*<.001)	1.18 (*p*=.244)		
Walking ability ^d^				
Pretest vs. posttest (1-week)	−34.22 (34.10)	−2.98 (25.20)	4.98	<.001
Pretest vs. posttest (1-month)	−51.42 (52.20)	−18.83 (29.33)	3.68	<.001
Pair *t* test	2.54 (*p*=.015)	4.07 (*p*<.001)		

^a^ Frail was measured by the Clinical Frail Scale; ^b^ Dyspnea was measured by the Modified Medical Research Council; ^c^ LEME was measured by the 30-second chair stand test; ^d^ Walking ability was measured by the 6-minute walking distance.

### GEE-Assessed Interactions Between Time and Frailty, Dyspnea, LEME, and WA at Baseline

GEE was used to control for confounding factors, including age, BMI, CCI, FEV1/FVC, gender, educational level, religion, smoking, drinking, and self-rated health status. At 1-week posttest, the reductions in frailty and dyspnea (−0.32 and −0.15, respectively) and increase in LEME (+0.78) were more significant in the experimental group than in the control group (*p*<.05). At 1-month posttest, these reductions (−0.30 and −0.48, respectively) and increase (+1.04), as well as an enhancement in WA by 18.17 m in the experimental group were all more significant than in the control group (*p*<.05). Thus, over time, the experimental group exhibited significantly greater changes in frailty, dyspnea, LEME, and WA than the control group (*p*<.05; Table 4).

**Table 4:** GEE Analysis of Group and Time Interactions of Frailty, Dyspnea, LEME and Walking Ability in Both Groups (*N*=91)

Variable	Beta	*SE*	95% CI		Wald χ^2^	*p*
			Lower	Upper		
Frailty						
Intercept	4.59	0.15	4.29	4.88	944.20	<.001
Group ^a^	0.12	0.23	0.33	0.58	0.29	.592
Time-posttest (1-week) ^b^	−0.32	0.08	−0.48	0.16	15.60	<.001
Time-posttest (1-month) ^c^	−0.30	0.10	−0.49	0.11	9.61	.002
Group×time (1-week) ^d^	−0.35	0.16	−0.67	0.03	4.54	.033
Group×time (1-month) ^e^	0.12	0.13	−0.13	0.37	0.87	.352
Dyspnea						
Intercept	2.98	0.08	2.81	3.14	1253.08	<.001
Group ^a^	0.18	0.14	−0.10	0.46	1.53	.217
Time-posttest (1-week) ^b^	−0.15	0.08	−0.30	0.00	4.11	.043
Time-posttest (1-month) ^c^	−0.48	0.09	−0.66	−0.30	27.69	<.001
Group×time (1-week) ^d^	−0.49	0.16	−0.80	−0.18	9.83	.002
Group×time (1-month) ^e^	−0.43	0.13	−0.68	−0.18	11.63	.001
LEME						
Intercept	6.20	0.54	5.14	7.25	133.32	<.001
Group ^a^	−0.15	0.84	−1.80	1.50	0.03	.857
Time-posttest (1-week) ^b^	0.78	0.26	0.27	1.30	8.89	.003
Time-posttest (1-month) ^c^	1.04	0.37	0.32	1.77	7.95	.005
Group×time (1-week) ^d^	−0.23	0.34	−0.90	0.45	0.43	.510
Group×time (1-month) ^e^	1.25	0.55	0.17	2.32	5.16	.023
Walking ability						
Intercept	135.87	13.53	109.35	162.39	100.80	<.001
Group ^a^	−8.89	21.13	−50.31	32.53	0.18	.674
Time-posttest (1-week) ^b^	2.98	3.68	−4.22	10.18	0.66	.418
Time-posttest (1-month) ^c^	18.17	3.92	10.49	25.85	21.51	<.001
Group×time (1-week) ^d^	31.24	6.55	18.40	44.09	22.74	<.001
Group×time (1-month) ^e^	37.42	7.60	22.51	52.32	24.21	<.001

*Note.* *SE*=standard error; CI=confidence interval.

^a^ The reference group was the control group; ^b^ The reference group was the control group, 1 week pretest; ^c^ The reference group was the control group, 1-month pretest; ^d^ (Posttest 1 week of experimental group vs. Pretest of experimental group) versus (Posttest 1 week of control group−Pretest of control group); ^e^ (Posttest 1 month of experimental group−Pretest of experimental group) versus (Posttest 1 month of control group vs. Pretest of control group.

## Discussion

### Demographic Analysis

The average age of the participants was 70 years. In general, they exhibited mild to moderate frailty, a CCI score >4.34, and multiple chronic diseases. Moreover, over half of the participants perceived their health status as poor. These findings align with Cachioni et al. (2021) and Pilleron et al. (2022), suggesting poor self-perceived health to be associated with increased risk of developing weakness and thus a significant indicator of frailness. However, clinical assessments have yet to integrate self-perceived health status into health evaluation measures.

The findings in this study related to high dyspnea levels, LEME performance, and compromised WA in older adults with COPD are similar to those of Torres-Sánchez et al. (2017). Increased levels of frailty, dyspnea, sarcopenia, muscle weakness, WA decline, anxiety, and depression during hospitalization are all associated with reduced motivation to participate in rehabilitation exercises (Maddocks et al., 2016). Hence, prolonged hospital stays increase the risk of falls and curtail lower limb mobility, increasing dependency (Torres-Sánchez et al., 2017). Also, the results of this study concur with Maddocks et al.'s and Torres-Sánchez et al.’s findings regarding the physiological and psychological effects of frailty on hospitalized individuals. However, frailty is not currently integrated into the COPD health assessment program, underscoring the need for early related interventions.

### Effectiveness Evaluation of PREP Intervention

In this study, the GEE findings indicate that improvements in frailty, dyspnea, LEME, and WA over time were significantly higher in the experimental group than the control group. The intervention effect of the 4-week PREP is consistent with that of a 6-week intervention also targeting muscle strength training conducted on patients with chronic lung disease reported by Mittal et al. (2015). This may be attributable to the incorporation by PREP of both pout breathing and inspiratory muscle strength training to ameliorate dyspnea. Beaumont et al. (2018) and Brighton et al. (2020) highlighted the enhancement effect of improved dyspnea on patient comfort and, subsequently, patient willingness to participate in comprehensive rehabilitation training. The results of this study indicate that, compared with those described in Mittal et al. (2015), the PREP program is better tailored to the needs of older adults with frailty and COPD.

Most empirical studies in the field of pulmonary rehabilitation have primarily focused on lower extremity muscle strength training for older adults with frailty and COPD (Mittal et al., 2015; Rutkowski et al., 2020; Torres-Sánchez et al., 2017). However, the symptoms of frailty often overlap with COPD symptoms (Proietti & Cesari, 2020). COPD patients experience chronic respiratory inflammation, leading to persistent symptoms like thick phlegm, wheezing, and dyspnea (Rahi et al., 2023). As dyspnea severity increases, the degree of frailty also tends to increase (Kennedy et al., 2019). In addition to lower extremity muscle strength training, PREP also includes a theoretically grounded back percussion component designed to enhance sputum clearance through pursed lip breathing and inspiratory muscle training with the goals of improved ventilation efficiency, increased oxygen concentration, better carbon dioxide elimination, and enhanced exercise capacity (Finamore et al., 2021). Therefore, in this study, the application of PREP was intended to improve or delay the progression of frailty. This approach not only emphasizes lower extremity muscle strength training but also underscores the significance of back percussion and diaphragm training.

In the EG, the PREP intervention improved frailty and dyspnea significantly as well as enhanced muscle endurance and walking distance. The GEE analysis showed that the mean frailty scores improved from 0.18 at baseline to 0.67 at 1 month, indicating that longer intervention duration was positively associated with greater frailty improvement. This finding aligns with the results reported by Maddocks et al. (2016). Also, this partially confirms the effectiveness of the PREP-designed exercise program in managing frailty in unstable patients with COPD. The PREP program was designed based on the characteristics of COPD and frailty symptoms (Beek et al., 2020) and was implemented and monitored in this study by discharge preparation service case managers. The PREP intervention used in this study was designed with reference to the exercise protocol proposed by Liao et al. (2015). It includes disease education, postural drainage and lung percussion, pursed-lip breathing, leg-raising exercises on the bed, and calf muscle strengthening. These exercises provide multiple benefits for pulmonary rehabilitation in patients with frailty and COPD, including reduced carbon dioxide retention, improved sputum clearance and respiratory function, enhanced muscle strength and cardiorespiratory endurance, and reduced dyspnea and fatigue, thereby alleviating frailty symptoms. Therefore, in summary, the PREP exercise program designed in this study, when properly implemented, is an effective option for treating frailty in hospitalized patients with frailty and COPD.

However, in terms of implementation, several challenges must be addressed to effectively expand the application of the PREP intervention in clinical practice and policy. These barriers include insufficient resources, inadequate staff training, and issues with patient adherence. Thus, it will be necessary to develop standardized training programs and integrate PREP into routine care activities and treatment plans. A team-based approach should be adopted to increase patient acceptance and participation in the exercise regimen. Also, at patient discharge, establishing regular follow-up visits will be crucial to ensuring patients continue to receive guidance and support after leaving the hospital.

### Limitations

In terms of methodology, the single-blind design used in this study may have introduced observer bias, potentially affecting the subjective evaluations of intervention effectiveness. Future research should consider using a double-blind design. Also, the recruitment of patients from a thoracic ward only may negatively influence the generalizability of the results. The efficacy of this intervention should be investigated in patients from nonrespiratory departments as well to enhance generalizability. Finally, in light of the challenges faced by patients in continuing the program after discharge, a remote rehabilitation exercise care model may be applied to sustain patient motivation to continue the exercise program at home.

### Conclusions

The positive influence of a 4-week PREP program on the health status of older patients with frailty and COPD, specifically in terms of frailty symptoms, dyspnea, LEME, and WA, was validated in this study. The findings support incorporating PREP into future pulmonary rehabilitation programs targeting patients with frailty and COPD and including PREP in routine nursing practice and health care policy. PREP may be implemented as a subacute health care model for the management of COPD-related debilitation in hospital settings. In addition, research should delve deeper into the long-term effects of PREP, considering its implementation in various health care settings, and evaluating its cost-effectiveness and enhancement effect on quality of life in this vulnerable patient population.

### Implications for Research and Practice

Frailty assessments should be part of the standard care provided to hospitalized elderly individuals with COPD. The PREP is unique and tailored to address the distinctive characteristics and debilitating symptoms associated with COPD. The exercise package includes process reengineering, breathing tools, health education charts, and the use of bedside equipment. In acute hospital settings, PREP should be considered for incorporation into the COPD patient care model to improve the quality of care provided to elderly patients with frailty and COPD.
